# Barrier-to-Autointegration Factor (BAF) involvement in prelamin A-related chromatin organization changes

**DOI:** 10.18632/oncotarget.6697

**Published:** 2015-12-20

**Authors:** Manuela Loi, Vittoria Cenni, Serena Duchi, Stefano Squarzoni, Carlos Lopez-Otin, Roland Foisner, Giovanna Lattanzi, Cristina Capanni

**Affiliations:** ^1^ CNR-National Research Council of Italy, Institute of Molecular Genetics, Unit of Bologna, 40136 Bologna, Italy; ^2^ Laboratory of Musculoskeletal Cell Biology, IOR, 40136 Bologna, Italy; ^3^ Osteoarticolar Regeneration Laboratory, Rizzoli Orthopaedic Institute, 40136 Bologna, Italy; ^4^ Department of Biomedical and Neuromotor Sciences (DIBINEM), University of Bologna, 40123 Bologna, Italy; ^5^ Department of Biochemistry and Molecular Biology, Medical Faculty, Oviedo University, 33006 Oviedo, Spain; ^6^ Max F. Perutz Laboratories, Medical University of Vienna, A-1030 Vienna, Austria

**Keywords:** prelamin A, barrier-to-autointegration factor, chromatin, Hutchinson-Gilford progeria syndrome, Nestor-Guillermo progeria

## Abstract

Chromatin disorganization is one of the major alterations linked to prelamin A processing impairment. In this study we demonstrate that BAF is necessary to modulate prelamin A effects on chromatin structure. We show that when prelamin A and BAF cannot properly interact no prelamin A-dependent effects on chromatin occur; similar to what is observed in human Nestor Guillermo Progeria Syndrome cells harboring a BAF mutation, in HEK293 cells expressing a BAF mutant unable to bind prelamin A, or in siRNA mediated BAF-depleted HEK293 cells expressing prelamin A. BAF is necessary to induce histone trimethyl-H3K9 as well as HP1-alpha and LAP2-alpha nuclear relocalization in response to prelamin A accumulation. These findings are enforced by electron microscopy evaluations showing how the prelamin A-BAF interaction governs overall chromatin organization. Finally, we demonstrate that the LAP2-alpha nuclear localization defect observed in HGPS cells involves the progerin-BAF interaction, thus establishing a functional link between BAF and prelamin A pathological forms.

## INTRODUCTION

BAF is a DNA binding protein that allows DNA filament interconnection *in vivo* and condensing of longer DNA molecules *in vitro* [1]. BAF localizes ubiquitously in cells, and several nuclear physiological events including post-mitotic nuclear assembly, chromatin remodeling, gene expression and DNA damage repair, seem to depend on proper BAF cellular distribution and expression [2], [3]. In the nucleus, BAF directly binds three fundamental groups of proteins: LEM-domain proteins [4–7], histones [8], [9] and nuclear lamins [10], [5]. Lamins are components of the nuclear lamina, a proteinaceous meshwork underlying the inner nuclear membrane. This structure arises from the polymerization of type V intermediate filaments encoded by the *LMNA* gene, named lamin A and lamin C, which, in combination with lamin B, provide a molecular link between the inner nuclear membrane and the genome. In particular, it has been demonstrated that components of the nuclear lamina directly interact with DNA and with proteins able to influence the accessibility to genetic information [11]. Thus, it is not surprising that a wide range of rare diseases, collectively named laminopathies, results from *LMNA* mutations. Muscular dystrophy, cardiomyopathy, neuropathy, lipodystrophy and progeroid syndromes are overlapping disorders identified in laminopathic patients [12]. At the molecular level, *LMNA* gene mutations affecting prelamin A processing lead to an acceleration in aging, causing adipose tissue atrophy, bone resorption and other systemic symptoms as described in Mandibuloacral Dysplasia (MAD), Hutchinson-Gilford Progeria Syndrome (HGPS), Familiar Partial Lipodystrophy type 2 (FPLD2) and Restrictive Dermophathy (RD) patients [12].

Prelamin A is the precursor of lamin A, and, unlike other types of lamins, it undergoes a specific maturation pathway. If maturation fails, prelamin A accumulation affects nuclear morphology [13], chromatin structure and DNA binding protein function [14–16] through a mechanism that is poorly understood.

We previously demonstrated *in vivo* molecular interaction between BAF and different prelamin A forms [17]. Prelamin A affects BAF cellular distribution inducing its nuclear localization; in accordance, prelamin A mutated forms identified in laminopathic cells have a similar effect [18]. Given that several chromatin modifying proteins have been identified among BAF binding partners [8], [9], [19], it is conceivable that the effects on chromatin organization caused by prelamin A could potentially depend on its interaction with BAF. The study reported here was aimed at demonstrating that the BAF-prelamin A interaction is necessary to mediate prelamin A accumulation effects on chromatin organization. To this end, we took advantage of Nestor-Guillermo Progeria Syndrome (NGPS) skin fibroblasts induced to accumulate prelamin A, and HEK293 cells transfected with prelamin A constructs in combination with different BAF mutants. NGPS is a rare progeroid syndrome characterized by aged appearance, growth retardation and decreased subcutaneous fat [20]. This disease is due to a point mutation (c.34G > A [p.Ala12Thr]) in the *BANF1* gene, codifying for BAF. In our experiments we observed that the expression of both NGPS-BAF mutant and a BAF mutant (BAF-G47E) unable to interact with the inner nuclear membrane components, affect the ability of prelamin A to modify chromatin organization. We demonstrate that the redistribution of histone H3 trimethylated at lysine 9 (H3K9m3), of HP1-alpha, and of the chromatin interacting protein LAP2-alpha, induced by prelamin A, need a proper prelamin A-BAF interaction. This is also required to preserve the overall prelamin A-dependent chromatin reorganization. In addition, we demonstrate the involvement of BAF in the deleterious effects triggered by progerin (a truncated prelamin A form accumulated in HGPS cells) on LAP2-alpha, observed in HGPS cells. Our results demonstrate a functional link between prelamin A and BAF allowing for a better understanding of the mechanism underlying pathological aging.

## RESULTS

### NGPS cells show dysmorphic nuclei with altered BAF, lamin A/C and prelamin A distribution which is associated with impaired prelamin A-mediated H3K9m3 intranuclear clustering

**Figure 1:**
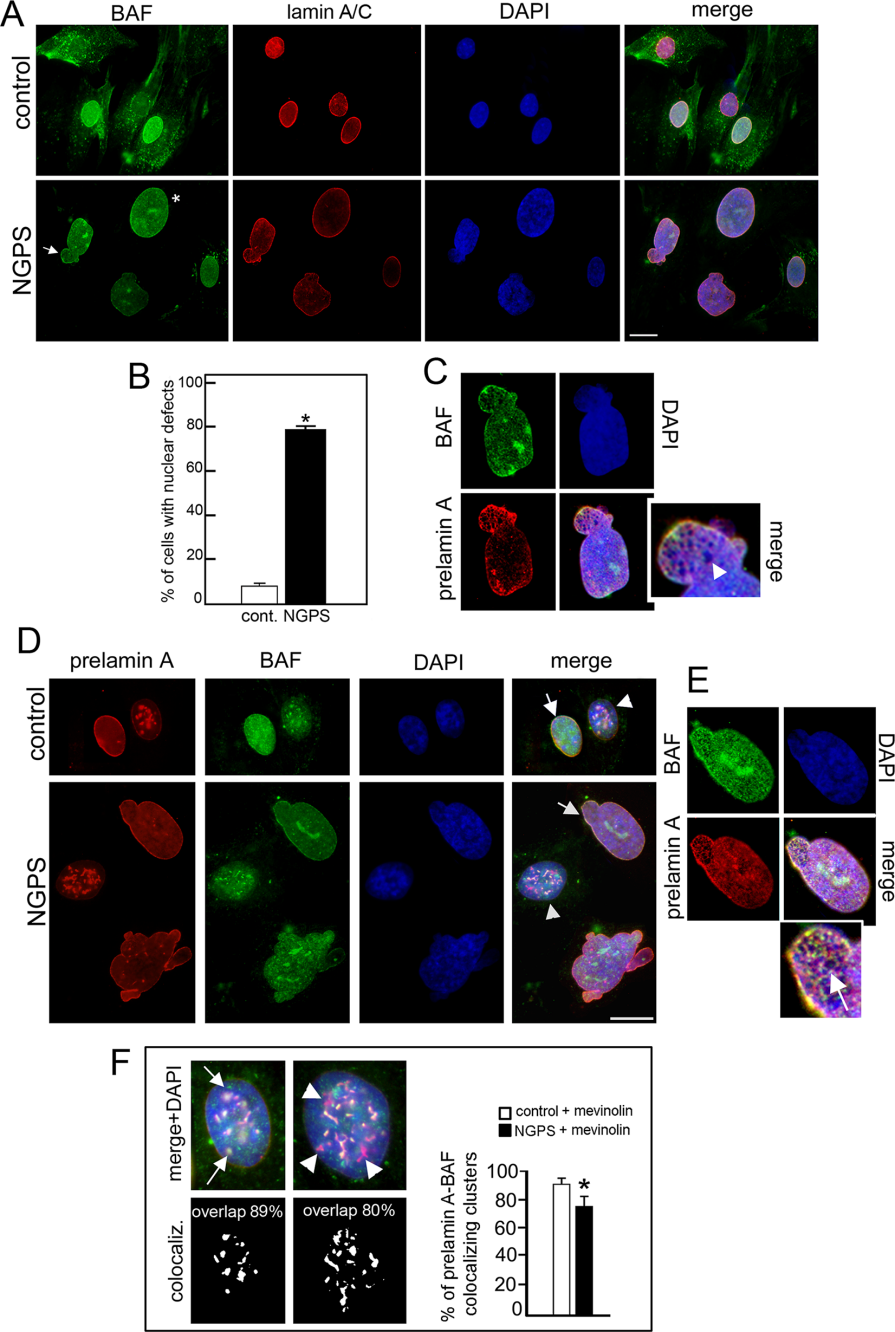
NGPS BAF mutation affects nuclear morphology, induces lamin A/C and prelamin A distribution defects (**A**) BAF (green) and lamin A/C (red) localization in control and Nestor-Guillermo Progeria Syndrome (NGPS) cells. Asterisk: nuclear enlargement, arrow: nuclear bleb. Nuclei are costained by DAPI. Bar 10 μm. (**B**) The percentage of cells showing nuclear alterations is reported in the graph as means ± S.D. of three different counts (100 nuclei per count). Asterisk: statistically significant difference at the Student's *t*-test (*P* < 0.05). (**C**) Higher magnification of NGPS nucleus indicated by arrow in A. Arrowhead: honeycomb. (**D**) Prelamin A (red) and BAF (green) immunofluorescence detection in control (cont.) and NGPS mevinolin treated cells. Arrows: prelamin A/BAF colocalization at the nuclear periphery. Arrowheads: nuclei with intranuclear prelamin A aggregates. DNA was stained by DAPI. Bar 10 μm. (**E**) Higher magnification of control (cont.) and NGPS nuclei with prelamin A intranuclear aggregates indicated by arrowheads in D. In a control nucleus prelamin A/BAF costained intranuclear aggregates are indicated by arrows while in NGPS nucleus prelamin A intranuclear aggregates missing BAF staining are indicated by arrowheads. Percentage of prelamin A/BAF overlapped aggregates is indicated. The percentages of prelamin A foci colocalized with BAF foci are indicated. At least 100 cells were analyzed for each experiment. Data are presented as mean ± S.D. (*P < 0.001). (**F**) Higher magnification of NGPS cell nucleus indicated by arrow in D. Arrow: honeycomb structures.

In accordance with previously described results [21], we observed that in NGPS cells the BAF-A12T mutation affected BAF protein level. BAF was detectable in NGPS nuclei but hardly visible in the cytoplasm. In control cells, BAF was present in both cellular compartments (Figure 1A). Lamin A/C staining highlighted NGPS nuclear morphology defects, as described [21]. Increase in nuclear size and/or presence of nuclear blebs were observed in 80% of *BANF1* mutated cells (Figure 1A asterisk and arrow, Figure 1B) while in control cells, less of 20% of nuclei were dysmorphic. Lamin A/C and BAF staining colocalized at the nuclear lamina of control and NGPS cells, however NGPS nuclei were characterized by honeycomb labeling patterns where rarefaction of Dapi staining was detectable (Figure 1C arrowhead).

In NGPS cells prelamin A processing is normal as demonstrated by the absence of prelamin A and progerin staining (Figure S1). Mevinolin addition to NGPS cells induced the accumulation of prelamin A at the nuclear lamina and at intranuclear aggregates, as observed in control cells. Prelamin A and BAF colocalized in control cells at both the nuclear periphery and the intranuclear aggregates (Figure 1D arrow and arrowhead, Figure 1E arrows). In contrast, in mevinolin-treated NGPS cells, though BAF nuclear recruitment was observed, BAF-prelamin A colocalization was impaired. In particular we observed that colocalization at intranuclear aggregates was partially lost (Figure 1D–1E arrowheads) while, at the nuclear lamina both proteins revealed a colocalization characterized by honeycomb pattern (Figure 1D–1F arrow).

**Figure 2:**
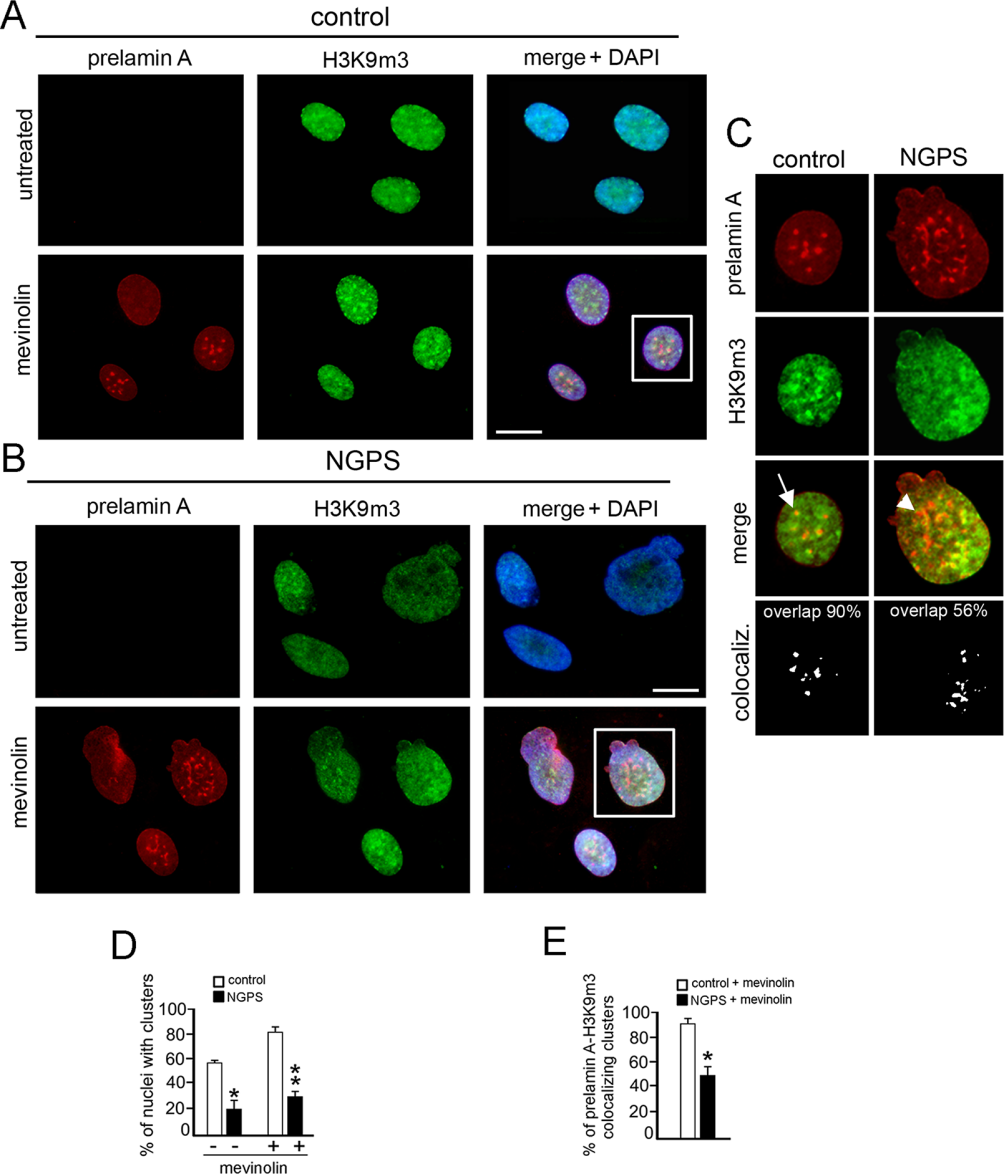
NGPS BAF mutation impairs prelamin A-related H3K9m3 intranuclear clustering (**A**) Prelamin A (red) and histone H3 trimethylated at lysine 9 (H3K9m3, green) localization in control fibroblasts untreated or treated with mevinolin. DNA staining with DAPI is shown in merge (merge + DAPI). Bar 10 μM. (**B**) Prelamin A (red) and H3K9m3 (green) localization in NGPS fibroblasts untreated or treated with mevinolin. DNA staining with DAPI is shown in merge (merge + DAPI). Bar 10 μM. (**C**) Higher magnification of nuclei showed in A and B (squares). Prelamin A (red), histone H3K9m3 (green) and merge without DAPI staining are shown. Arrow: prelamin A-H3K9m3 costained intranuclear aggregate; arrowhead: prelamin A intranuclear aggregate in which H3K9m3 staining is missing. The percentage of prelamin A-H3K9m3 colocalized spots is reported. (**D**) Statistical analysis. The graph shows the percentage of nuclei showing clustered H3K9m3 in control and NGPS cells. White bars: control cells untreated (−) or mevinolin treated (+); black bars: NGPS cells untreated (−) or mevinolin treated (+). Values are means of two independent experiments ± S.D.; for each experiment a total of 100 nuclei were considered. (*P < 0.004), (**P < 0.001). (**E**) The percentages of prelamin A foci colocalized with H3K9m3 are indicated. Values are means of two independent experiments ± standard deviation. At least 100 cells were analyzed for each experiment. (*P < 0.001).

Prelamin A accumulation in mevinolin-treated cells induced H3K9m3 clustering in more than 80% of control cells in which the intranuclear aggregates of lamin A precursor perfectly colocalized with H3K9m3 aggregates (Figure 2A–2B, 2E and 2D arrow). On the contrary, in NGPS cells, even if prelamin A was accumulated and normally distributed in the nucleus, H3k9m3 clustering failed (Figure 2B and 2C) and only a limited number of prelamin A intranuclear aggregates (50%) colocalized with H3k9m3 aggregates (Figure 2D arrowhead and 2E). However, it is important to note that in NGPS untreated cells, H3K9m3 was barely detectable and less than 20% of nuclei showed a normal clustered distribution, while in control cells a normal pattern was detectable in 60% of cells (Figure 2D and 2C).

### Prelamin A-BAF interaction is compromised by the *BANF1* gene mutation occurring in NGPS cells

Since in NGPS cells prelamin A and BAF colocalization appears partially lost, we wondered if prelamin A-BAF binding could be compromised by the BAF-A12T mutation. To this aim, HEK293 cells were transfected with FLAG-tagged lamin A (LA-WT) or FLAG-tagged prelamin A (LA-C661M) in combination with three GFP-tagged BAF constructs: wild type BAF (BAF-WT), NGPS-BAF mutant (BAF-A12T) and a BAF mutant unable to interact with nuclear envelope proteins (BAF-G47E) [17], [18], [8], [22]. Since, we previously demonstrated, in accordance with other authors [23] and [24], that the overexpression of LA-WT causes not only lamin A increase but also a definite level of prelamin A accumulation [17], [18], we harvested transfected cells 24 hour post-transfection. In this way, prelamin A protein amount in LA-WT expressing cells is lower than lamin A [17], [18].

**Figure 3:**
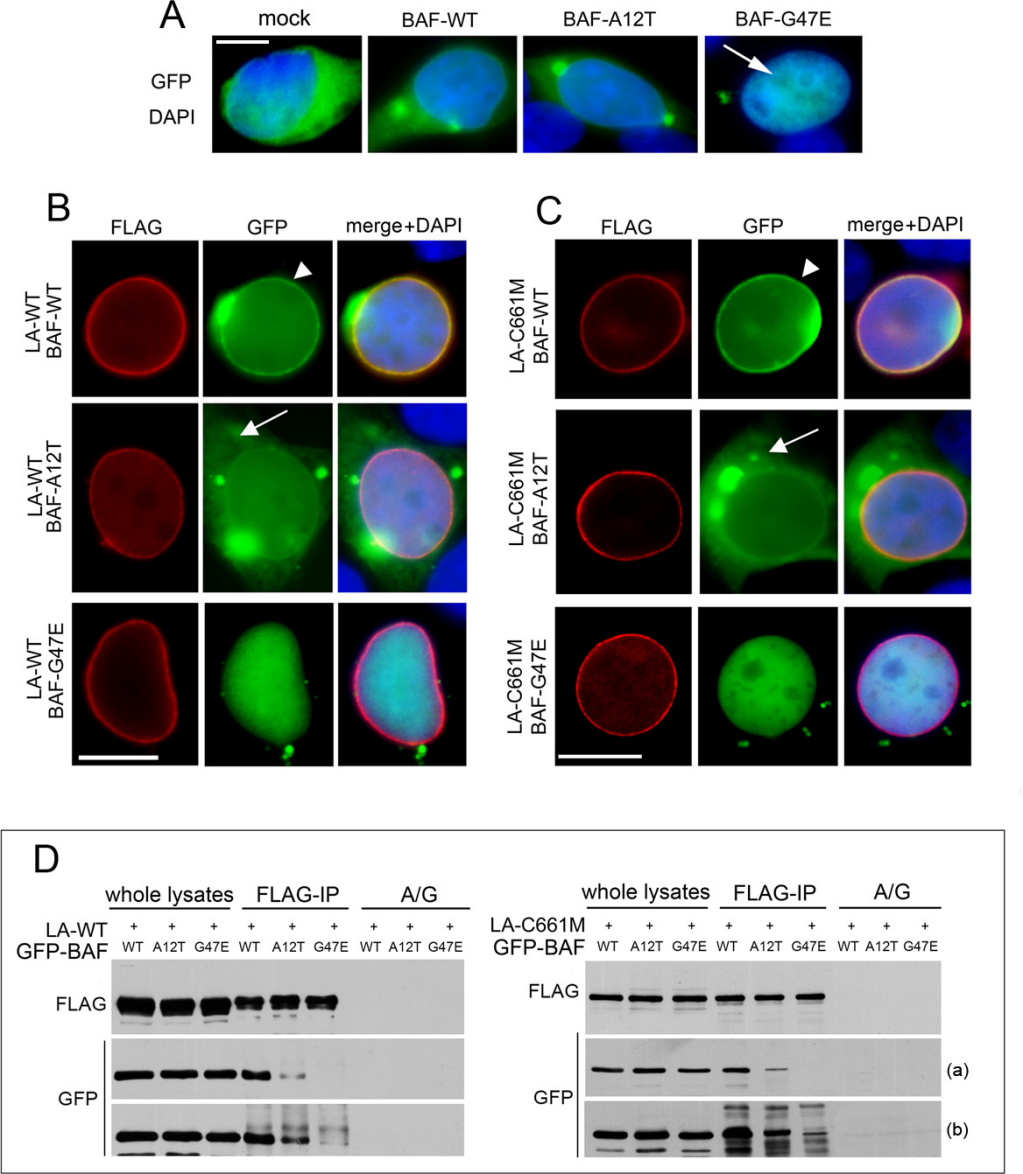
Evaluation of BAF mutants localization and interaction with prelamin A (**A**) GFP-BAF mutants localization in HEK293 cells. GFP-empty vector (mock), BAF-WT, BAF-A12T and BAF-G47E localization in combination with DAPI staining is shown. Bar 10 μM. (**B**) GFP-BAF-mutants localization in FLAG-tagged wild type lamin A (LA-WT) expressing cells. (**C**) GFP-BAF-mutants localization in FLAG-tagged prelamin A (LA-C661M) expressing cells. In B and C FLAG staining (red), GFP signal (green). Arrowheads: nuclear lamina GFP localization; arrows: cytoplasmic GFP distribution. DAPI staining is shown in merge. Bar 10 μM. Images shown are representative of 80% of transfected cells. (**D**) Coimmunoprecipitation study performed in HEK293 cells transfected with LA-WT or LA-C661M in combination with GFP-BAF constructs. Western blotting analysis of FLAG and GFP-tagged proteins in whole lysate and coimmunoprecipitated complexes (FLAG-IP) are shown. No proteins staining was observed in absence of anti-FLAG antibody (A/G). Two different exposures, short (a) and long (b), are shown.

The localization and expression of GFP-tagged proteins were evaluated (Figure 3A and Figure S2A). BAF-WT and BAF-A12T showed a similar cytoplasmic/nuclear distribution while BAF-G47E localized exclusively in the nucleoplasm (Figure 3A arrow). The expression of BAF-WT in combination with LA-WT or LA-C661M induced BAF-WT nuclear recruitment (Figure 3B–3C arrowheads) [17]. On the contrary, BAF-A12T mutant localization was slightly affected by expression of FLAG-tagged lamins. In particular, cytoplasmic GFP-BAF-A12T staining was still detectable (Figure 3B–3C arrows and Figure S4) and the nuclear rim signal increase was very low. As expected, BAF-G47E did not change at all its nucleoplasmic distribution (Figure 3B–3C and Figure S4). The analysis of the fluorescence intensity profile confirmed BAF-A12T and BAF-G47E translocation defects (Figure S4). Western blotting analysis showed that exogenous proteins were expressed at similar levels and at the predicted molecular weight (Figure S2A).

Anti-FLAG immunoprecipitation experiments performed in cotransfected cells showed a minor BAF-A12T interaction with both LA-WT and LA-C661M compared with BAF-WT recovery. As expected, a faint band corresponding to BAF-G47E was observed only after a long exposure time (Figure 3D).

### Prelamin A induces HP1-alpha and LAP2-alpha nuclear relocalization by interacting with BAF

Since the BAF mutation occurring in NGPS cells not only compromises prelamin A-BAF interaction but also impairs prelamin A-mediated H3K9m3 intranuclear recruitment, we wondered if this last alteration could also be caused by anomalous interaction of prelamin A and BAF. To this aim we evaluated the effect of prelamin A on chromatin marker distribution in models where prelamin A and BAF fail to interact properly.

The expression of prelamin A is reported to induce HP1-alpha clustering without any effects on protein level [25, 26]. A similar result was obtained in HEK293 expressing prelamin A constructs (Figure S3). On the contrary, HP1-alpha localization was unaffected by the expression of GFP-empty vector or GFP-BAF constructs (Figures S2B and 4A).

**Figure 4:**
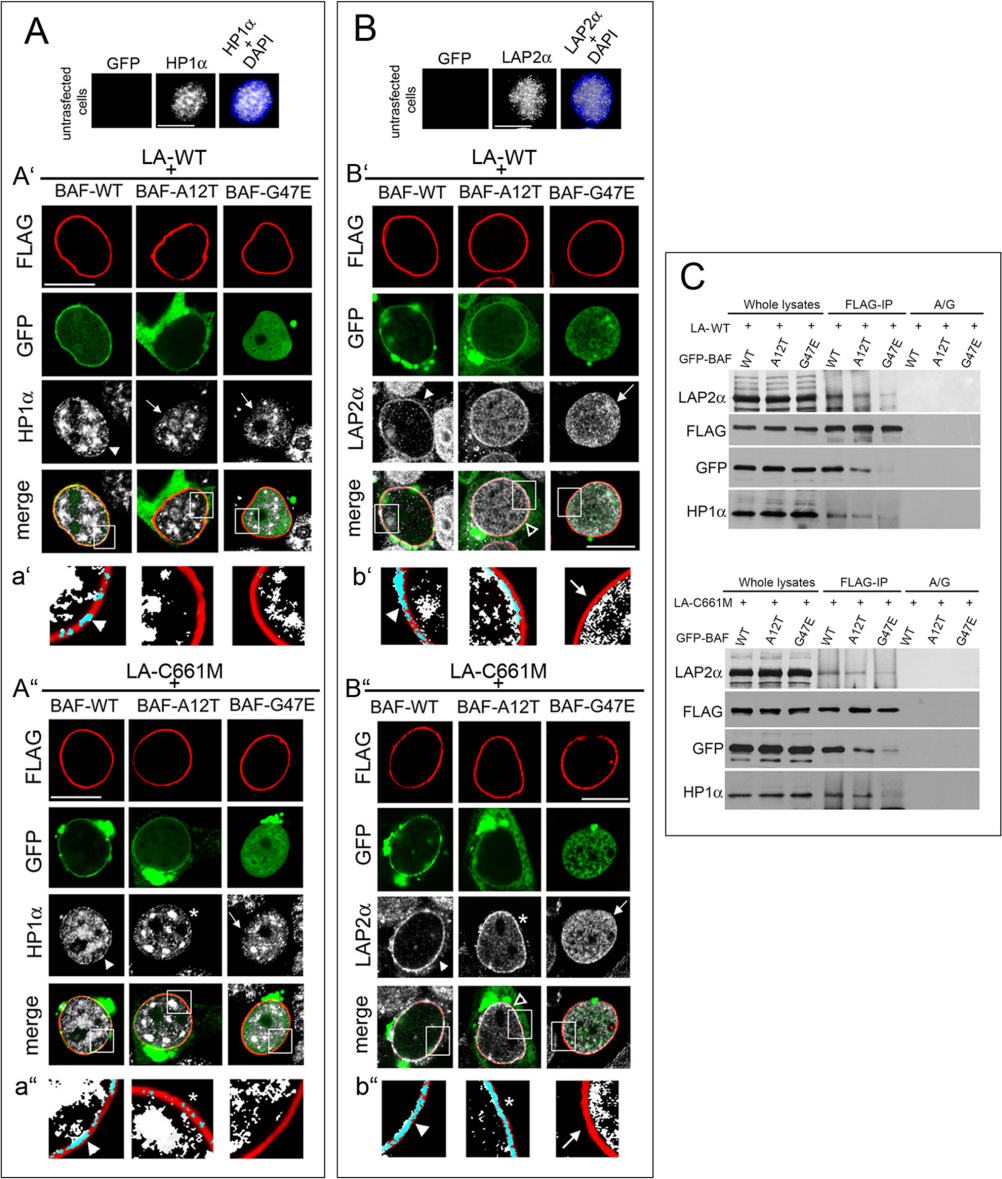
BAF governs HP1-alpha and LAP2-alpha nuclear relocalization in prelamin A accumulating cells (**A**) HP1-alpha (HP1α) localization in HEK293 cells expressing FLAG-tagged lamin constructs in combination with GFP-BAF mutants. Top line: HP1α nuclear distribution in untransfected cells. HP1α staining (grey) is combined with DAPI (HP1α+DAPI). Bar 10 μM. A’) HP1α localization in cells expressing wild type lamin A (LA-WT+) in combination with BAF-WT or BAF-A12T or BAF-G47E. FLAG staining (red), GFP (green), HP1α (grey). Bar 10 μM. a’) Density dot plot of FLAG and HP1α colocalization staining showed in A'. FLAG (red), HP1α (white), merge (blue). Arrowhead: FLAG/HP1α colocalization. A”) HP1α localization in HEK293 cells expressing prelamin A (LA-C661M+) in combination with BAF-WT, BAF-A12T, BAF-G47E. FLAG staining (red), GFP (green), HP1α (grey). Bar 10 μM. a”) Density dot plot of FLAG and HP1α colocalization staining showed in A″. FLAG (red), HP1α (white), merge (blue). Arrowhead and asterisk indicate FLAG/HP1α colocalization. In panel (A') and (A″) arrowheads indicate HP1α nuclear periphery localization increase relative to untransfected cells (shown in top line); arrows: HP1α intranuclear localization pattern; asterisk: partial HP1α nuclear lamina localization pattern. Images shown are representative of 80% transfected cells. (**B**) LAP2-alpha (LAP2α) localization in HEK293 cells expressing FLAG-tagged lamin constructs in combination with GFP-BAF mutants. In the top line LAP2α nuclear distribution in untransfected cells. LAP2α staining (grey) is combined with DAPI (LAP2α+DAPI). Bar 10 μM. B’) LAP2α localization in cells expressing wild type lamin A (LA-WT+) in combination with BAF-WT or BAF-A12T or BAF-G47E. FLAG staining (red), GFP (green), LAP2*α* (grey). Bar 10 μM. b’) Density dot plot of FLAG and LAP2α colocalization staining shown in B'. FLAG (red), LAP2*α* (grey), merge (blue). Arrowhead: FLAG/LAP2*α* colocalization. Arrow: absence of LAP2α staining at the nuclear periphery. (B”) LAP2α localization in HEK293 cells expressing FLAG-tagged wild type prelamin A (LA-C661M+) BAF-WT or BAF-A12T or BAF-G47E. FLAG staining (red), GFP (green), LAP2*α* (grey). Bar 10 μM. b”) Density dot plot of FLAG and LAP2α colocalization shown in B″. FLAG (red), LAP2α (white), merge (blue). Arrowhead; FLAG/LAP2α colocalization. Asterisk: partial FLAG/LAP2α colocalization. Arrow: absence of LAP2α staining at the nuclear periphery. In (B') and (B″) arrowheads indicate LAP2α nuclear periphery localization, arrows indicate LAP2α nucleoplasmic distribution, empty arrowheads indicate a partial LAP2α nuclear lamina recruitment, asterisk indicate a more detectable LAP2α nuclear lamina staining observed in LA-C661M+BAF-A12T expressing cells. Images are representative of 80% transfected cells. (**C**) LA-WT and LA-C661M coimmunoprecipitation experiment performed in cotransfected cells. Protein complexes recovered by anti-FLAG antibody (FLAG-IP) as well as whole lysates were subjected to FLAG, GFP, HP1α and LAP2α Western blotting detection. No protein bands were observed in protein A/G negative control (A/G).

In cells expressing prelamin A constructs (LA-WT and LA-C661M) in combination with BAF-WT, HP1-alpha nuclear periphery recruitment was detectable (Figure 4A’–4A” and 4a’–4a” arrowheads) while in cells expressing prelamin A constructs in combination with BAF mutants, the effect on HP1-alpha was impaired (Figure 4A’–4A” arrows and 4a’–4a”). However, in LA-C661M/BAF-A12T expressing cells, a barely detectable HP1-alpha staining was perceptible at the nuclear rim (Figure 4A” and 4a” asterisks). Western blotting analysis showed that this result was not related to protein amount or molecular weight changes (Figure S2B).

To strengthen our finding, we evaluated also LAP2-alpha [25]. This nucleoplasmic protein interacts with intranuclear lamins, and the accumulation of prelamin A modifies its localization [25]. In cells expressing prelamin A constructs alone or in combination with BAF-WT, we observed that LAP2-alpha was located at the nuclear periphery (Figure S3 asterisk and Figure 4B’–4B” and 4b’–4b” arrowheads). On the contrary, in untransfected cells or in cells expressing singularly any BAF construct, LAP2-alpha nucleoplasmic localization did not change (Figure 4B and Figure S2C). Interestingly, a decrease in prelamin A ability to modify the nuclear distribution of LAP2-alpha was observed when prelamin A constructs were expressed in combination with BAF mutants (Figure 4B’–4b’ and 4B”–4b”). The nuclear lamina recruitment of LAP2-alpha was abolished by BAF-G47E co-expression (Figure 4B’–4b’ and 4B”–4b”’ arrows), but it still partially occurred when cotransfected with BAF-A12T (Figure 4B empty arrowheads). In particular, we observed that the BAF-A12T inhibitory effect on LAP2-alpha nuclear periphery repositioning was more evident when LA-WT was coexpressed (Figure 4B’–4b’). Differently, LA-C661M coexpression left a faint LAP2-alpha nuclear rim staining (Figure 4B”–4b” asterisks). Western blotting analysis showed that the LAP2-alpha protein level was not affected by exogenous protein expression (Figure S2C). In addition, the evaluation of fluorescence intensity profiles confirmed these results (Figure S4). A coimmunoprecipitation study performed in cotransfected cells demonstrated that BAF mutants were able to decrease prelamin A interaction with LAP2-alpha and HP1-alpha (Figure 4C). These results are in accordance with the known preferential BAF interaction with prelamin A [17], [18]; indeed, prelamin A (LA-C661M) partially affects chromatin organization (HP1-alpha and LAP2-alpha recruitment at the nuclear periphery) also when BAF-prelamin A binding is compromised (prelamin A/BAF-A12T complex). On the contrary, in lamin A (LA-WT) expressing cells the prelamin A protein amount seems to be insufficient, although increased, to overcome the impairment of prelamin A/AF-A12T interaction.

**Figure 5:**
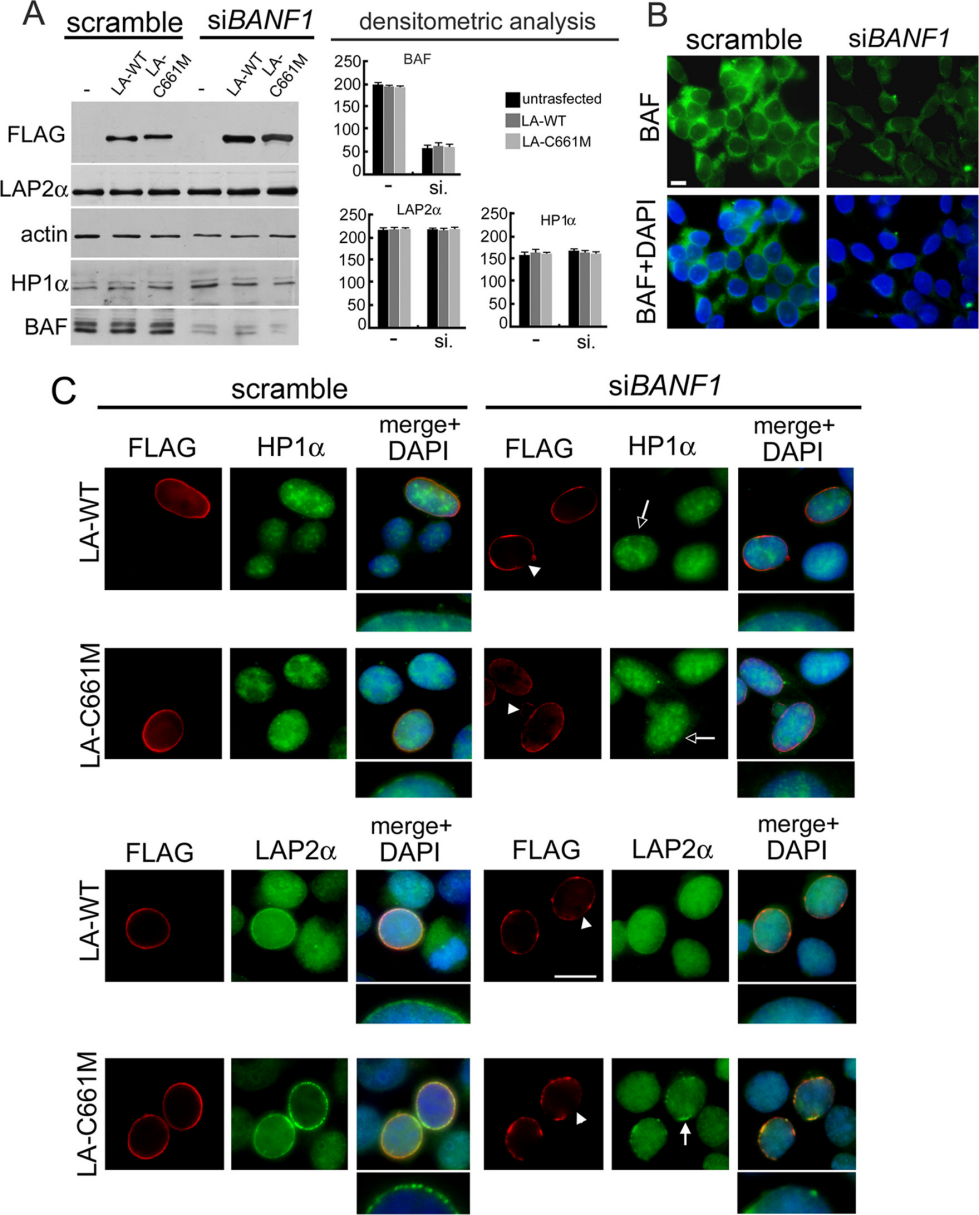
BAF depletion interferes with prelamin A-mediated HP1-alpha and LAP2-alpha nuclear relocalization (**A**) Western blotting evaluation of total lysates from HEK293 cells subjected to BAF depletion by siRNA treatment. Cells treated with scramble or *BANF1* siRNA (si*BANF1*) were untransfected (−) or transfected with LA-WT or LA-C661M constructs. FLAG, LAP2α, HP1α and BAF proteins bands are shown. Actin: protein loading control. Western blotting densitometric analysis is reported. (**B**) Immunofluorescence evaluation of BAF in HEK293 cells treated with scramble or *BANF1* siRNA. BAF (green) and DNA staining (DAPI) are shown. Bar 10 mm. (**C**) Immunofluorescence detection of LA-WT, LA-C661M, HP1α and LAP2α in cells treated with scramble siRNA or si*BANF1*. FLAG (red), HP1α and LAPα (green). DNA was costained by DAPI and shown in merge. Arrowheads: nuclear lamina rarefaction staining of FLAG tagged proteins. Empty arrows: absence of the HP1α nuclear periphery staining increase. Arrow indicates the defective LAP2α nuclear periphery recruitment. Higher magnification of nuclear periphery distribution of HP1α and LAP2α staining, merged with DAPI, is shown. Bar 10 μm.

We confirmed our results in BAF-siRNA treated HEK293 cells expressing prelamin A constructs (Figure 5). BAF reduction (Figure 5A–5B) elicited LA-WT and LA-C661M localization defects (Figure 5C arrowheads). As already observed in NGPS cells, transfected nuclei were characterized by FLAG staining rarefaction at the nuclear lamina. In accordance, the prelamin A mediated effects on HP1-alpha and LAP2-alpha nuclear localization are undetectable (in the case of HP1-alpha) (Figure 5C empty arrows) or partially detectable (in the case of LAP2-alpha) (Figure 5C). In particular, some nuclear periphery LAP2-alpha aggregates were observed in LA-C661M expressing cells (Figure 5C arrow), however a complete nuclear lamina recruitment of LAP2-alpha without nucleoplasmic staining was never observed. HP1-alpha, LAP2-alpha and FLAG tagged protein amounts were unaffected by si*BANF1* silencing (Figure 5A).

### Prelamin A-BAF interaction affects chromatin organization

**Figure 6:**
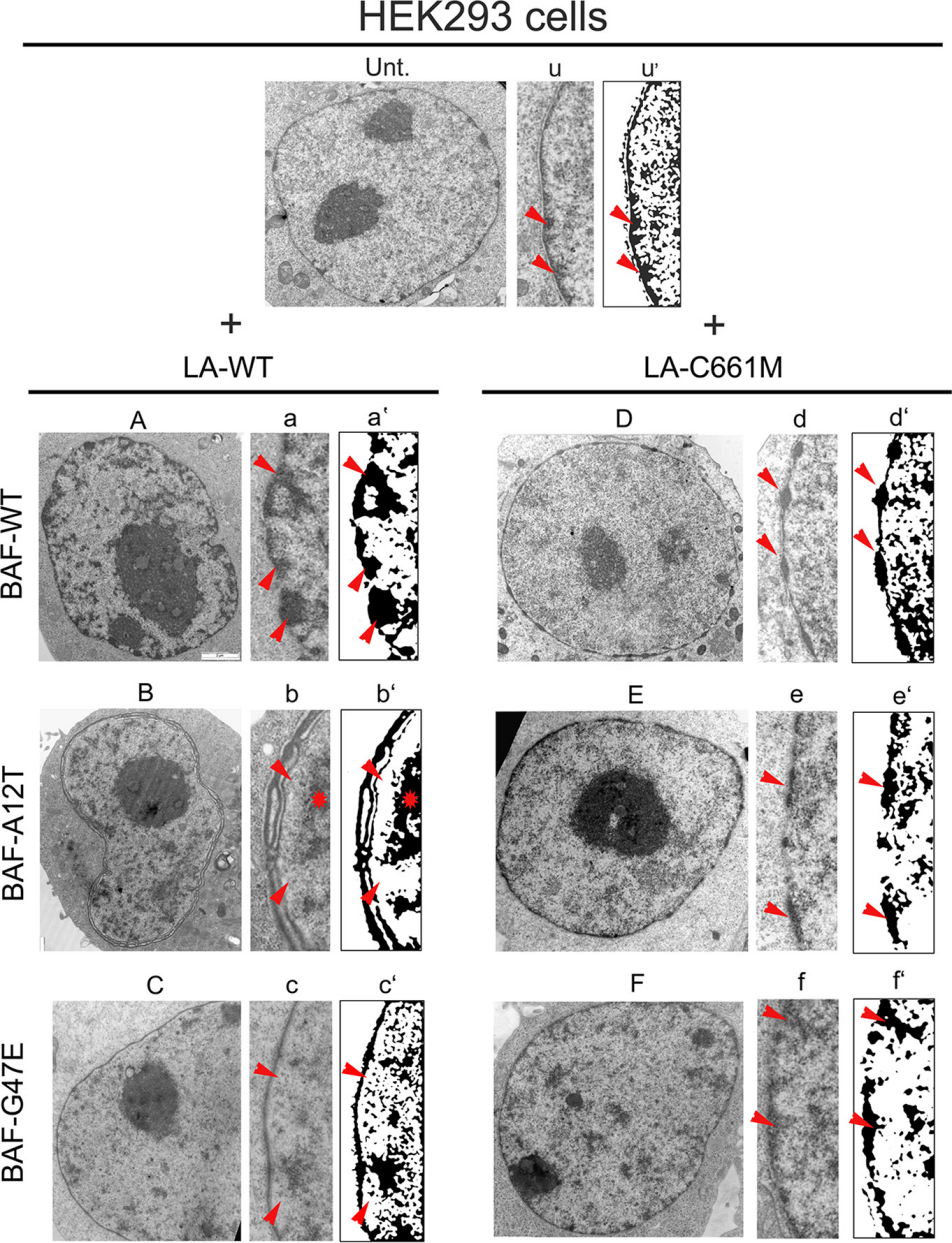
Electron microscopy evaluation of prelamin A/BAF complex effects on chromatin organization Electron microscopy evaluation of HEK293 nuclei in untransfected (Unt.) or prelamin A and BAF constructs cotransfected cells. (Unt.) Ultrastructural organization of untransfected HEK293 cell nucleus. (u) Enlargement of nuclear periphery. (u’) Density dot plot of picture showed in (u). Red arrowheads: peripheral heterochromatin. (**A**) Nucleus of cell expressing LA-WT in combination with BAF wild BAF-WT. (a) Enlargement of nuclear periphery. (a’) Density dot plot of picture showed in (a). Red arrowheads: heterochromatin. (**B**) Nucleus of cell expressing LA-WT in combination with BAF-A12T. (b) Enlargement of nuclear periphery. (b’) Density dot plot of picture showed in (b). Red arrowheads: peripheral heterochromatin detachment. Red star: detached heterochromatin clump. (**C**) Nucleus of cell expressing LA-WT in combination with BAF-G47E. (c) Enlargement of nuclear periphery. (c’) Density dot plot of picture showed in (c). Red arrowheads: heterochromatin rarefaction. (**D**) Nucleus of cell expressing LA-C661M in combination with BAF-WT. (d) Enlargement of nuclear periphery. (d’) Density dot plot of picture showed in (d). Red arrowheads: “beads-on-a-string appearance” heterochromatin. (**E**) Nucleus of cell expressing LA-C661M in combination with BAF-A12T. (e) Enlargement of nuclear periphery. (e’) Density dot plot of picture showed in (e). Arrowheads: heterochromatin. (**F**) Nucleus of cell expressing LA-C661M in combination with BAF-G47E. (f) Enlargement of nuclear periphery. (f’) Density dot plot of picture showed in (f). Red arrowheads: heterochromatin. Representative images of chromatin organization observed in 70% of analyzed nuclei, for each cotransfection experiment, are shown.

To evaluate the influence of BAF on chromatin organization due to prelamin A, we performed an electron microscopy study on HEK293 cells expressing FLAG-tagged proteins in combination with BAF constructs. Untransfected cells showed a heterochromatin layer at the nuclear periphery, underlying the normally organized nuclear envelope (Figure 6 Unt.-u-u’ arrows). Cells expressing LA-WT in combination with BAF-WT showed a normal chromatin organization with increase of heterochromatin clusters located at the nuclear periphery (Figure 6A–6a–6a’ arrows).

On the contrary, when LA-WT was expressed in combination with BAF-A12T, the peripheral heterochromatin did not adhere to the nuclear lamina. In addition, the architecture of the nuclear wall was affected, as an aberrant duplication of the nuclear lamina and nuclear envelope was often detectable (Figure 6B–6b–6b’ star/arrows).

The coexpression of LA-WT with BAF-G47E had no effect on chromatin organization. The ultrastructural appearance of HEK293 nuclei expressing this construct combination was similar to that observed in untransfected cells, even thought a mild-grade heterochromatin loss with nuclear lamina detachment was observed (Figure 6C–6c–6c’ arrows).

The combination of LA-C661M with BAF-WT induced the recruitment of heterochromatin clumps at the nuclear lamina with an impressive beads-on-a-string appearance (Figure 6D–6d–6d’ arrows). Interestingly, the combination of LA-C661M with BAF-A12T or BAF-G47E impeded the beads-on-a-string chromatin clustering while some heterochromatin clustering was observed (Figure 6E–6e–6e’ arrows and 6F–6f–6f’ arrows).

### BAF mediates LAP2-alpha nuclear relocalization caused by progerin

**Figure 7:**
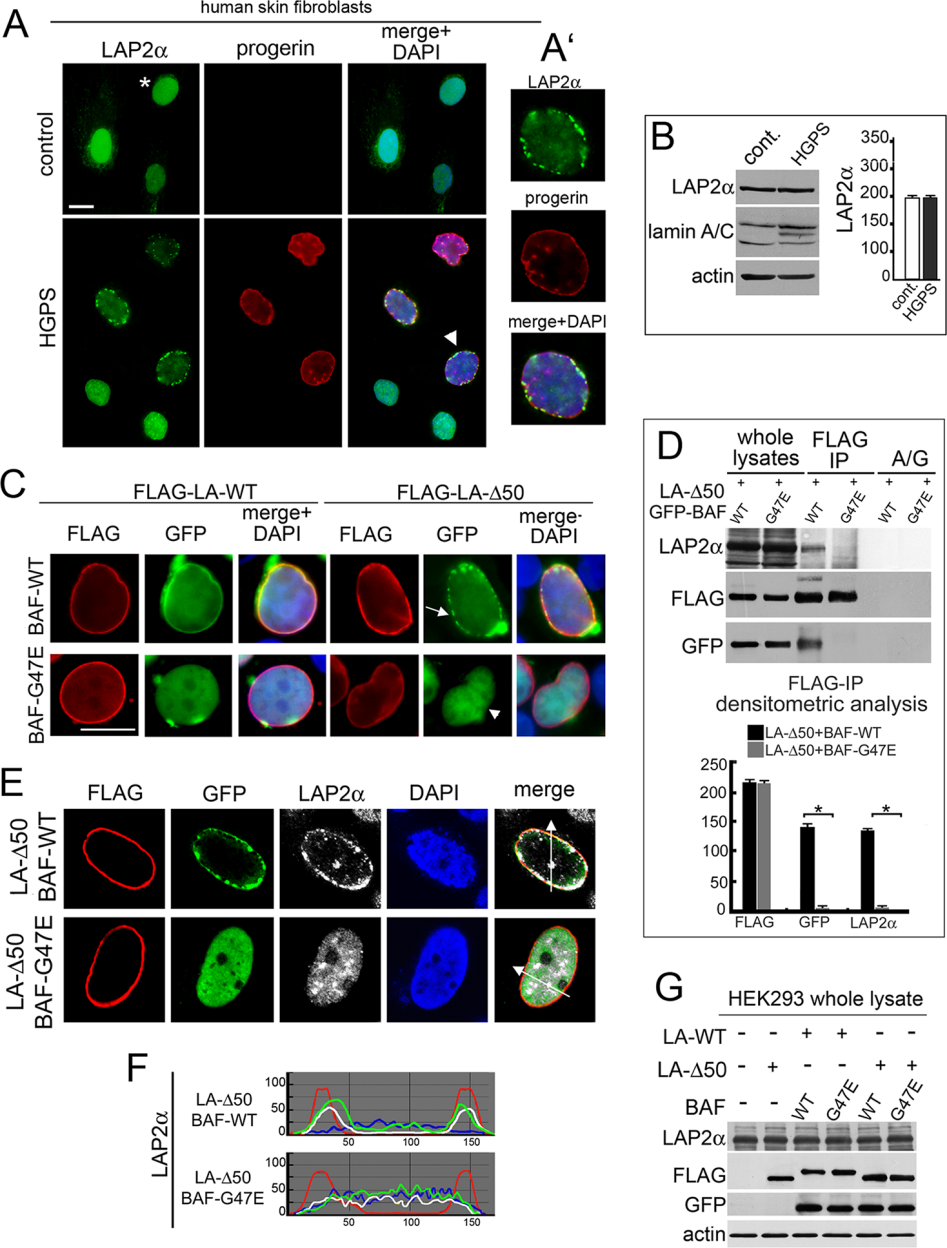
BAF is involved in the LAP2-alpha nuclear distribution observed in progerin expressing cells (**A**) Localization of LAP2α and progerin in control and HGPS cells. LAP2α (green), progerin (red). DNA staining with DAPI is showed in merge. Bar 10 μm. Asterisk: LAP2α nucleoplasmic distribution. Arrowhead: HGPS nucleus shown at higher magnification in A'. (**B**) Western blotting evaluation of LAP2α and lamin A/C in control and HGPS cells. Actin: protein loading control. Densitometric analysis of LAP2α immunoblotted bands was performed in triplicate experiments. (**C**) BAF-WT and BAF-G47E localization in cells expressing LA-WT or progerin construct (FLAG-LA-Δ50). FLAG (red), GFP (green). DNA staining with DAPI is shown in merge. Bar 10 μm. Arrow: BAF-WT nuclear periphery aggregates. Arrowhead: BAF-G47E nucleoplasmic localization. Images are representative of 80% of transfected cells. (**D**) Western blotting evaluation of coimmunoprecipitation performed in cotransfected cells. FLAG, GFP and LAP2α immunoblotted bands obtained from whole lysates and anti-FLAG immunoprecipitation (FLAG-IP) samples are shown. No protein bands were detected in protein A/G (A/G) negative control. Densitometric analysis of FLAG, GFP and LAP2α immunoblotted bands is shown. Values are means of three independent experiments ± S.D. (*P < 0.001). (**E**) LAP2α nuclear distribution in cells coexpressing LA-Δ50 and BAF-WT or progerin unbinding mutants BAF-G47E. FLAG (red), GFP (green), LAP2α (grey). DNA staining performed with DAPI is excluded in merge. Images shown are representative of 75% of cotransfected cells. (**F**) Fluorescence intensity profile along arrows drawn in (E). The top graph indicates intensity profile of BAF-WT (green), LA-Δ50 (red), LAP2α (white) and DAPI (blue). The bottom graph indicates intensity profile of BAF-G47E (green), LA-Δ50 (red), LAP2α (white) and DAPI (blue). (**G**) Western Blotting evaluation of LAP2α in HEK293 cells untransfected or transfected with LA-WT or LA-Δ50 in combination with wild type BAF (WT) or (G47E). Immunoblotted bands obtained from detection of whole lysates are shown. LAP2α, FLAG, GFP. Actin: protein loading control.

Considering that progerin, a mutated form of prelamin A accumulated in HGPS cells, interacts with BAF, we wondered if the progerin-BAF complex could affect LAP2-alpha nuclear distribution in these cells. To this aim, we first evaluated LAP2-alpha localization and expression in cells from patients affected by Hutchinson-Gilford Progeria Syndrome (Figure 7A). Using methanol fixation, we could unveil a peculiar LAP2-alpha distribution linked to progerin accumulation [27]. In control cycling cells, LAP2-alpha nucleoplasmic localization was observed (Figure 7A asterisk). In contrast, progerin increase in HGPS cells reduced intranuclear LAP2-alpha staining and induced a clustered nuclear lamina localization (Figure 7A arrowhead and Figure 7A’) bearing perfect colocalization with progerin. LAP2-alpha nuclear redistribution was not related with its amount (Figure 7B).

**Figure 8:**
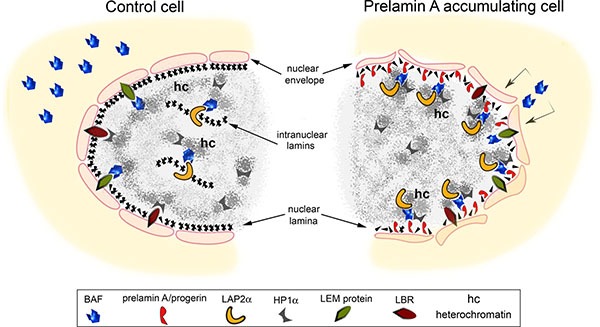
Speculative cartoon of the mechanism involving prelamin A-BAF interaction in chromatin organization change In normal cells BAF localizes both at the cytoplasm and in the nucleus. At the nuclear level BAF is distributed close to the inner nuclear membrane where it interacts with LEM-proteins (LAP2, emerin and MAN1) as well as in the nucleoplasm where it binds intranuclear form of LAP2: LAP2α. Lamin B receptor (LBR) and heterochromatin binding protein 1 alpha (HP1α) are involved in the organization of heterochromatin. The accumulation of prelamin A/progerin affects BAF localization in the cell influencing chromatin organization. In particular, the accumulation of lamin A precursors induce BAF translocation from the cytoplasm to the nuclear lamina where prelamin A and BAF interact. BAF mediates prelamin A accumulation effects on chromatin organization. Prelamin A takes advantage of BAF interaction with DNA and DNA organizing proteins to modify chromatin organization as demonstrated by LAP2α and HP1α nuclear relocalization.

For evaluating BAF involvement in progerin-mediated effects on LAP2-alpha localization, we expressed FLAG-tagged progerin (FLAG-Δ50) in HEK293 cells (Figure S3). Endogenous BAF recruitment at the nuclear rim of FLAG-Δ50 expressing cells was observed, but aggregates were formed (Figure S3 asterisk). When GFP-tagged BAF-WT was expressed in combination with FLAG-Δ50 a similar distribution was observed (Figure 7C arrow), interestingly FLAG-Δ50 was not able to recruit the BAF-G47E mutant at the nuclear lamina (Figure 7C arrowhead).

Coimmunoprecipitation studies revealed that FLAG-Δ50 was able to bind BAF-WT but failed to bind BAF-G47E mutant (Figure 7D). More interestingly, BAF-G47E was able to abrogate FLAG-Δ50 influence on LAP2-alpha nuclear localization without causing effects at the protein level (Figure 7E–7F–7G).

## DISCUSSION

Chromatin disorganization is one of the main characteristics of laminopathies with prelamin A processing defects. Accumulation of different prelamin A forms highly modifies the structural and genetic organization of the nucleus [11], and pharmacological approaches reducing the prelamin A protein level can at least in part limit deleterious effects on nuclear functions [28–30].

Thus, the identification of prelamin A-binding proteins able to influence chromatin organization by connecting prelamin A with chromatin, is a major goal, in order to develop to intervening therapies to contrast the damage induced by prelamin A.

In this regard, we previously identified BAF as a possible prelamin A molecular mediator involved in chromatin organization defects which characterize prelamin A linked diseases [18]. In our previous study we demonstrated *in vivo* prelamin A-BAF interaction and, more interestingly, we observed that BAF was retained in the nucleus in all prelamin A-accumulating diseases [17], [18] suggesting a functional link between prelamin A and BAF. Since a human BAF proteome analysis revealed 56 high-confidence targets in a single cell type, including multiple proteins that regulate histone modifications [9] and because BAF both stabilizes the nuclear lamina structure and influences chromatin organization [19], we wondered if BAF could be a possible prelamin A modulator. The study we report here on prelamin A accumulation effects on chromatin organization in NGPS cells lends evidence to this hypothesis (Figure 7). In particular we observe that A12T-mutated BAF not only affects nuclear morphology, but leads to lamin A/C localization defects and, more interestingly, interferes with the typical H3K9m3 intranuclear clustering induced by non-farnesylated prelamin A accumulation. In NGPS cells lamin A biogenesis is normal and mevinolin administration results in prelamin A increases both at the nuclear lamina and at intranuclear aggregates, as expected. Nevertheless, the expression of prelamin A and its colocalization with BAF-A12T and H3K9m3 at intranuclear aggregates appear anomalous, suggesting that the integrity of the BAF-prelamin A protein platform could be affected by the BAF-A12T mutation, which, in turn, also leads to functional impairment of prelamin A mediated chromatin organization. In accordance, we observe that though the BAF-A12T mutation does not affect BAF dimerization or BAF binding to either DNA or emerin [21], its ability to interact with lamin A and prelamin A is reduced. Thus, in NGPS cells the decrease in prelamin A capacity to recruit BAF in the nucleus combined with a reduction in the amount of BAF protein [21] could damage the function of the protein platform governed by prelamin A.

It has been previously described that BAF is necessary for proper lamin A/C localization [31], that lamin A and prelamin A bind BAF directly [32], [10] and that lamin A and BAF influence each other's localization [33], [31]. In the absence of lamin A/C, BAF localizes exclusively in the cytoplasm and seems to be unable to translocate into the nucleus. BAF has a reciprocal influence on lamin A/C localization. Consistent with this notion, we observed lamin A/C honey-comb structures in NGPS cells. Therefore, it is conceivable that the decrease in both lamin A/BAF capability to interact reciprocally and in the BAF protein level observed in NGPS cells, mimic BAF depletion causing a secondary lamin A misfunction. On the other hand, secondary lamin A defects could further worsen BAF defects in NGPS trough a feedback loop. In addition, the NGPS-BAF mutation impedes the proper localization of emerin. In NGPS cells emerin loses its nuclear distribution and is found predominantly in the cytoplasm [21]. Interestingly, emerin interacts with prelamin A *in vivo* [34] suggesting that the NGPS-BAF mutation could perturb the prelamin A-BAF platform by affecting the binding partners needed for proper localization of prelamin A. Indeed emerin is necessary for the intranuclear localization of prelamin A aggregates, as demonstrated by prelamin A mislocalization observed in emerin null cells treated to accumulate lamin A precursor [34].

The pathogenic BAF mutation leads to nuclear misshaping, increased size, lobulation and blebbing [21]. In analogy with genetic disorders due to mutations in the nuclear lamina and nuclear envelope components, we observe that such irregular nuclei are characterized by abnormalities in chromatin organization related to epigenetic changes such as trimethylation of histone H3 at Lys9 and Lys27, histone H4 at Lys20 and the acetylation of histone H4 at Lys16 [35–37]. NGPS skin fibroblasts are characterized by loss of H3K9me3 which becomes more evident when prelamin A is accumulated. The decrease of H3K9me3 staining observed in NGPS cells is in accordance with the proposed BAF epigenetic function [9]. BAF interacts with nucleosomes and directly binds histone H3 and H4 influencing nucleosome accessibility to chromatin or, alternatively, recruiting specific regulators to chromatin [19]. In particular BAF interacts with the dimethyltransferase G9a [19]. This enzyme is responsible for H3-K9 dimethylation, a secondary histone modification necessary to silence chromatin via Suv39 h1 action. Interestingly BAF overexpression, as well as prelamin A accumulation, increases trimethylation status of histone H3 on lysine 9 [19]. In NGPS cells, such an epigenetic marker is strongly reduced and prelamin A accumulation fails to affect H3K9me3 intranuclear localization. These findings suggest that the BAF-A12T mutation could perturb the epigenetic function of BAF affecting its ability to interact with histone H3 or with enzymes involved in histone H3 secondary modification.

We confirmed our results in NGPS cells using an experimental model. Specific markers related to chromatin organization and dynamics, HP1-alpha and LAP2-alpha [25], were evaluated under conditions of impaired prelamin A-BAF interaction. We expressed prelamin A in combination with BAF-G47E, a BAF mutant whose ability to interact with histone H1/H3, inner nuclear membrane proteins (emerin and MAN1) [8] and prelamin A (this paper) is severely reduced. We observed that the expression of prelamin A constructs in combination with BAF-WT induced localization of BAF at the nuclear lamina, reorganization of HP1-alpha distribution and LAP2-alpha recruitment to the nuclear periphery, while in combination with BAF-A12T or BAF-G47E, decreased or abolished, respectively, BAF localization to the nuclear lamina as well as both HP1-alpha and LAP2-alpha relocalization in the nucleus were decreased or abolished respectively. Notably, in HGPS cells, recruitment of LAP2alpha to the nuclear periphery is mediated by WT-BAF and does not occur in the presence of BAF mutants. These findings allowed us to speculate about the possible molecular mechanism involved in progeroid syndromes linked to prelamin A processing defects and BAF mutations.

Aging is a complex process in which the most prominent hallmark is the accumulation of various types of DNA damage; among these, DSBs (DNA Double-Strand Breaks) are the most deleterious [38, 39]. In addition, aging brings about dramatic changes in the packaging of DNA into higher-order chromatin structures [40]. Perhaps the most significant of these changes are both the global loss of highly condensed, transcriptionally silent heterochromatin [41] and the alteration in histone composition during replicative aging [42–44]. In progeroid syndromes, due to prelamin A processing impairment, heterochromatin loss and a deficiency in DNA-repair processes have been described [45], [46].

Interestingly BAF binding with proteins involved in the DNA damage response has been reported [9]. In NGPS cells, BAF mutation could interfere directly with such functions while in HGPS cells the persistence of BAF-progerin binding could impair BAF interactions or slow down the dynamics of BAF protein complexes. In this regard, it has been recently demonstrated that prelamin A accumulation affects the mobility of the BAF binding protein HP1-alpha following DNA damage, strengthening its nuclear matrix association [47]. The delay in γ-H2AX foci formation demonstrates that the persistence of an heterochromatin status impedes DNA damage repair [47].

We observed that BAF is necessary to mediate both prelamin A and progerin effects on LAP2-alpha nuclear distribution. LAP2-alpha interacts with High Mobility Group nucleosome-binding proteins (HMGN) 5 [48]. The HMG class of proteins affects chromatin-related processes such as transcription, replication, and repair [49] causing changes in chromatin structure or at the level of histone post-translational modifications. LAP2-alpha is able to influence the nuclear distribution and mobility of HMGN5 [48], suggesting that this LEM-protein could indirectly affect the overall chromatin organization and influence the activation of specific DNA sequences.

Thus, prelamin A and progerin accumulation could also modify HMGN5 distribution across the genome through the control of LAP2-alpha nuclear localization, influencing gene expression in a pro-aging way [48].

However, further studies need to be performed in order to unveil the nuclear pathway specifically affected by the impairment of BAF function and, more interestingly, identify those common molecular events that enhance aging progression in NGPS and progeroid laminopathies.

## MATERIALS AND METHODS

### Cell cultures, transfection and siRNA

Skin fibroblast cultures were obtained from skin biopsies of healthy, NGPS [21] HGPS (culture passage numbers 10–15) [41] and RD (culture passage numbers 12–16) [50] patients following a written consent. Cultured were established and cultured in Dulbecco's modified Eagle's medium supplemented with 10% fetal calf serum (FCS) and antibiotics. For transfection experiments HEK293 cells were used. GFP-BAF and the mutated constructs BAF-A12T, BAF-G47E, full length FLAG-tagged prelamin A (LA-WT, pCI mammalian expression vector) and the mutated construct LA-C661M were transiently transfected into HEK293 cells using FuGene6 reagent (Roche). The transfection efficiency was over 75%. Biochemical and immunofluorescence analysis were performed 24 h after transfection. BAF depletion by siRNA treatment (Santa Cruz biotechnology SC-44804) was performed according to the manufacturer's protocol. In human skin fibroblast cells, the accumulation of prelamin A was obtained using 25 μM mevinolin (Sigma) in growth medium for 18 h.

### Western blotting and immunoprecipitation

For Western blotting evaluation HEK293 untransfected or transfected cells were processed in lysis buffer containing 20 mM Tris-HCl, pH 7.5, 1% SDS, 1 mM Na3VO4, 1 mM PMSF, 5% β-mercaptoethanol and protease inhibitors. Protein were subjected to SDS gradient gel (5–20%) electrophoresis and transferred to nitrocellulose membrane overnight at 4°C. Incubation with primary antibodies was performed for the indicated time. Bands were revealed by the Amersham ECL detection system. For coimmunoprecipitation experiments in order to evaluate tagged proteins interaction (FLAG-lamin A constructs and GFP-BAF constructs), HEK293 transfected cells were lysed in a buffer containing 50 mM TRIS-HCl, pH 8.0, 150 mM NaCl, 1% NP40, 0.1% SDS and protease inhibitors (IP-buffer). Lysates were incubated with specific antibodies overnight at 4°C. Control immunoprecipitation were performed in the presence of aspecific immunoglobulins. After the addition of 30 μl of protein A/G (Santa Cruz Biotechnology) for 45 min at 4°C, immunoprecipitated protein complexes were washed, added to Laemmli's buffer, boiled and subjected to western blot analysis. To evaluate Lap2-alpha and Hp1-alpha interaction with lamin A/BAF protein platform a previously described methods was used [25]. Briefly, HEK 293 transfected cells were fixed in 1% formaldehyde in PBS1X for 10 min at room temperature, and then lysed in buffer containing 1% SDS, 10 mM EDTA, 50 mM Tris-HCl pH 8.0 plus protease inhibitors. Cells were lysed by glass beads (Sigma) and thereafter by sonication. Immunoprecipitation was performed overnight at 4°C. Immunoprecipitated protein complexes were washed and subjected to Western blot analysis.

### Immunofluorescence

Human fibroblast and HEK293 transfected cells grown on coverslips were fixed in 4% paraformaldehyde at 4°C for 10 min and permeabilized with 0.15% Triton X-100 for 5 min and in methanol at −20°C for 7 minutes. Samples were incubated with PBS containing 4% BSA to saturate non-specific binding and incubated with primary antibodies and secondary antibodies. The nuclei were then counterstained with 4, 6-diamino-2-phenylindole (DAPI). The slides were mounted with an anti-fade reagent in glycerol and observed. Immunofluorescence microscopy was performed using a Nikon E600 epifluorescence microscope and a Nikon oil-immersion objective [100x magnification, 1, 3 NA (numerical aperture)]. Photographs were taken using a Nikon digital camera (DXm) and NIS-Element AR software. Confocal imaging was performed using a laser-scanning motorized confocal system (Nikon A1R, Nikon, Amsterdam, Netherlands) equipped with an Eclipse Ti-E inverted microscope and four laser lines (405, 488, 561, and 638 nm). Images were acquired with a Nikon 100x PlanApo Oil 1.4 NA oil objective. For each type of quantification, laser intensities and camera settings were maintained identically within the same experiment to allow comparison of different cell expressing constructs. Localization, colocalization and mean intensity analysis were performed using NIS-Element AR. Images were processed using Adobe Photoshop (Adobe Systems).

### Electron microscopy

Pellets from HEK293 transfected cells were fixed with 2.5% glutaraldehyde 0.1 M cacodylate buffer, pH 7.3. After post-fixation with 1% osmium tetroxide (OsO4) in cacodylate buffer for 1 h, pellets were dehydrated in an ethanol series, infiltrated with propylene oxide and embedded in Epon resin. Ultrathin sections were stained with uranyl acetate and lead citrate (15 min each) and were observed with a Jeol Jem-1011 transmission electron microscope, operated at 100 kV. At least 100 nuclei per sample were observed.

### Antibodies

The antibodies employed for western blot analysis or immunofluorescence labeling were: anti-FLAG, mouse monoclonal (Sigma M2, diluted 1:300, 1 h, for the immunofluorescence analysis and 1:1000, 1 h, for the Western blot analysis); anti-BAF, rabbit polyclonal (Santa Cruz Biotechnology FL-89, diluted 1:10, overnight at 4°C for immunofluorescence analysis; anti-GFP rabbit polyclonal (Santa Cruz Biotechnology FL, diluted 1:1000 for 1 h, for the Western blot analysis); anti-prelamin A, goat polyclonal (Santa Cruz Biotechnology SC-6214, used 1:100 overnight at 4°C for the immunofluorescence analysis) anti-lamin A/C, goat polyclonal (Santa Cruz Biotechnology N-18, used 1:100 overnight at 4°C, for the Western blot analysis); anti-progerin (Merck Millipore clone 13A4 diluted 1:10, overnight at 4°C for immunofluorescence analysis); anti-actin, goat polyclonal (Santa Cruz Biotechnology I-19, diluted 1:1000 for 1 h, for the Western blot analysis);anti-trimethyl-H3K9, rabbit polyclonal (Upstate, used 1:100 for 1 h at room temperature for the immunofluorescence analysis); anti-LAP2α, rabbit polyclonal Dechat [51] (used at 1:500 dilution for the immunofluorescence analysis and at 1:1000 dilution for the Western blot analysis overnight at 4°C); anti-HP1α, rabbit polyclonal (Upstate, used 1:500 for 1 h, for the Western blot analysis); anti-HP1α, rabbit polyclonal (Abcam, used at 1:100 dilution for immunofluorescence analysis).

## SUPPLEMENTARY MATERIALS FIGURES


