# Supplementary material for: Barrier-to-Autointegration Factor (BAF) involvement in prelamin A-related chromatin organization changes
Source: Oncotarget. 2015 Dec 20;7(13):15662–77. doi: 10.18632/oncotarget.6697 (PMC4941268; doi:10.18632/oncotarget.6697)
Supplement: Supplementary file 1 [file oncotarget-07-15662-s001.pdf]

Barrier-to-Autointegration Factor (BAF) involvement in prelamin A-related chromatin organization changes

Supplementary Materials

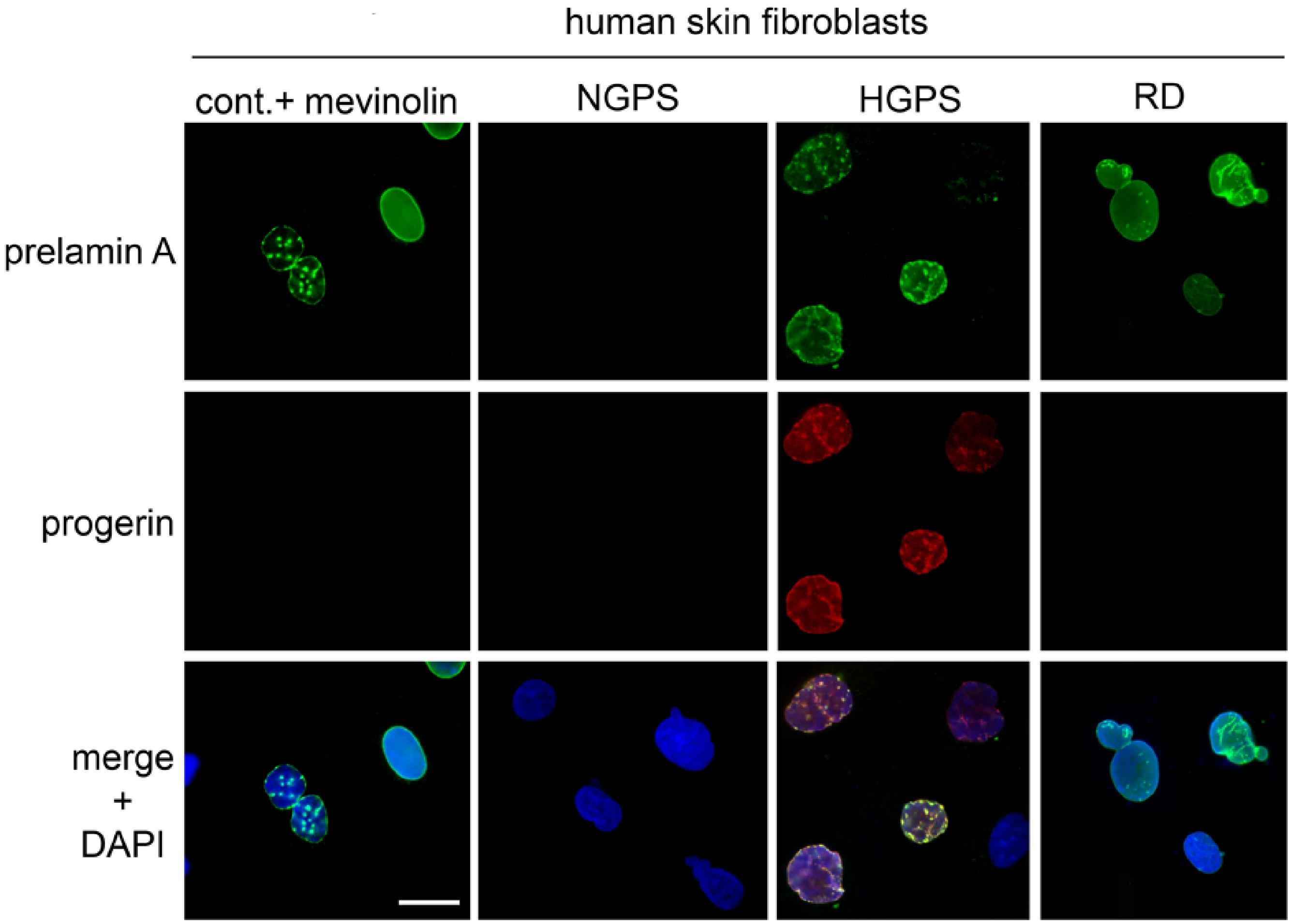

Supplementary Figure S1: Prelamin A and progerin evaluation in NGPS cells. Prelamin A (green) and progerin (red) immunofluorescence evaluation in skin fibroblasts from Nestor-Guillermo Progeria Syndrome (NGPS), Hutchinson-Gilford Progeria Syndrome (HGPS), Restrictive Dermophaty (RD) patients and control cells treated with mevinolin (cont. + mevinolin). Prelamin A and progerin immunofluorescence staining were merged with DAPI (merge + DAPI). Bar 10 µm.

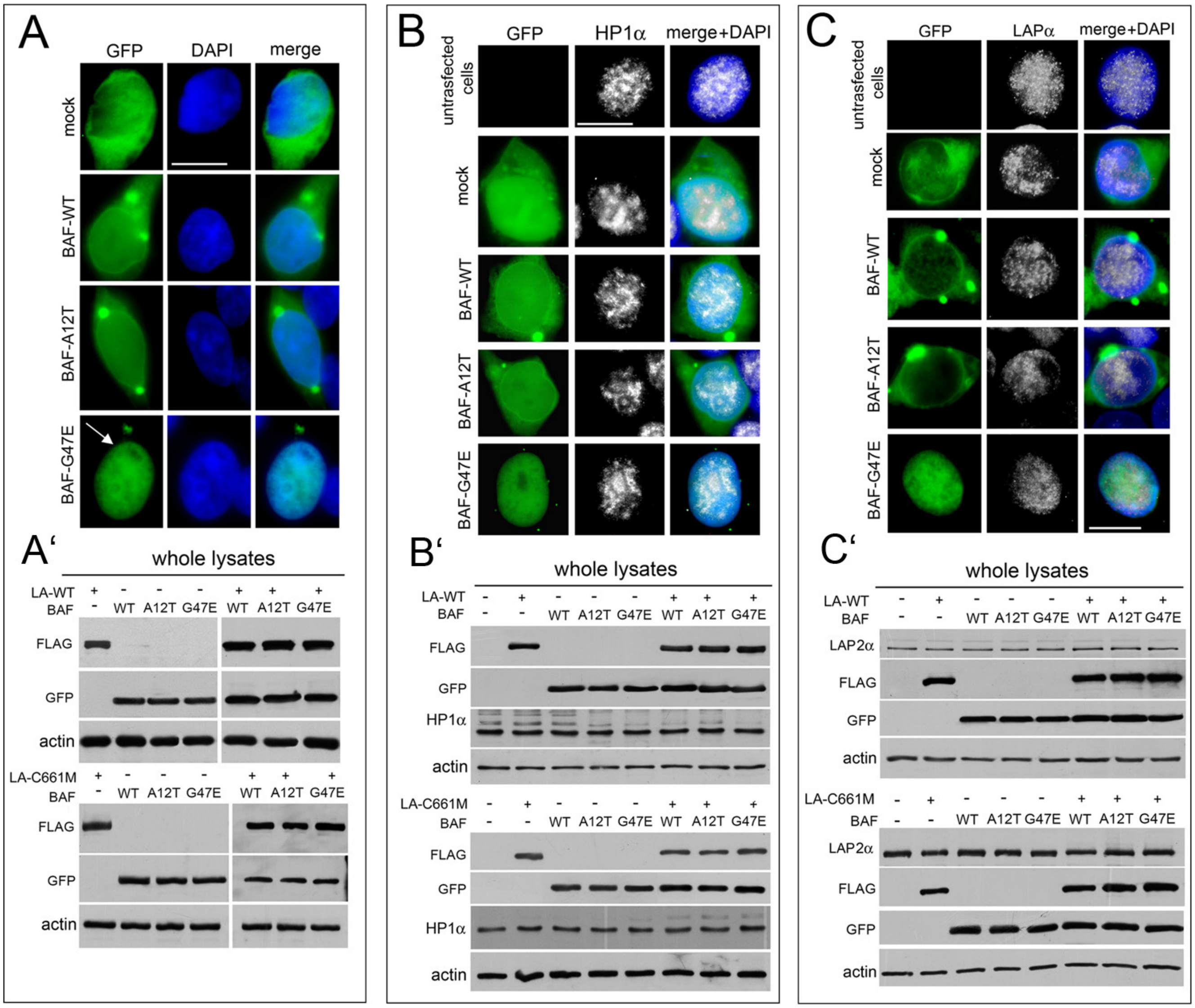

Supplementary Figure S2: GFP-BAF mutants localization and expression in HEK293 cells. Evaluation of effects on HP1-alpha and LAP2-alpha localization and FLAG-tagged proteins expression. (**A**) GFP-empty vector (mock), BAF-WT, BAF-A12T and BAF-G47E localization in HEK293 cells. Arrow: nucleoplasmic distribution of BAF-G47E mutant. GFP (green), DNA staining (DAPI) and merge are shown. Bar 10 μM. A') Western blotting analysis of FLAG and GFP-tagged proteins in total lysates of HEK293 cells expressing GFP-tagged proteins or FLAG-tagged proteins in a single or double transfection experiments. Actin: protein loading control. (**B**) HP1α localization in GFP-empty vector (mock), BAF-WT, BAF-A12T and BAF-G47E expressing cells. GFP (green), HP1-alpha (grey) and DNA staining (DAPI) are merged and shown in the right column. Bar 10 μM. B') Western blotting analysis of HP1α in HEK293 in untransfected cells and in cells expressing GFP-tagged or FLAG-tagged proteins, in a single and double transfection experiments. Actin: protein loading control. (**C**) LAP2α localization in GFP-empty vector (mock), BAF-WT, BAF-A12T, and BAF-G47E expressing cells. GFP (green), LAP2α (grey) and DNA staining (DAPI) are merged and shown at the right column. Bar 10 μM. C') Western blotting analysis of LAP2α, and FLAG immunoblotted total lysates of untransfected cells and cells expressing GFP-tagged proteins or FLAG-tagged proteins in a single and double transfection experiments. Actin: protein loading control.

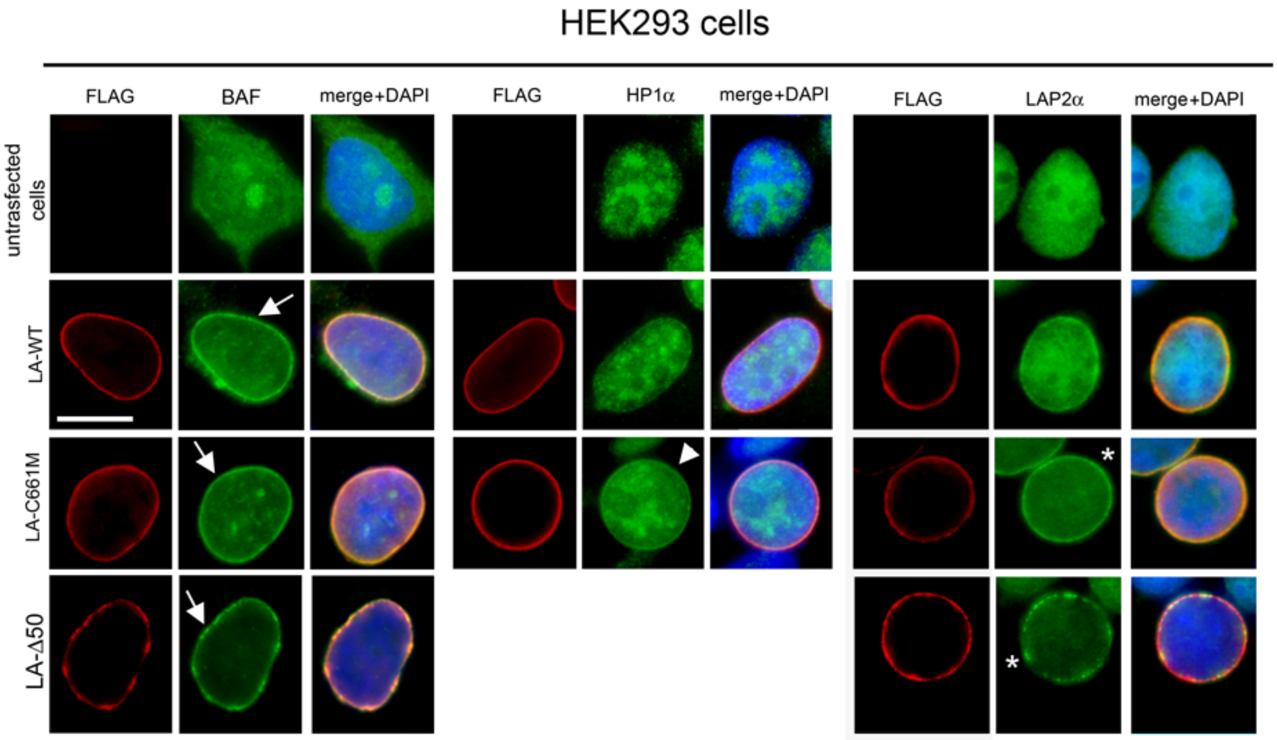

Supplementary Figure S3: Localization of endogenous BAF, HP1-alpha and LAP2-alpha in HEK293 cells expressing prelamin A constructs. Immunofluorescence evaluation of endogenous BAF, HP1α and LAP2α in HEK293 cells transfected with lamin A constructs. FLAG-tagged protein (red) are: wild type lamin A (LA-WT), prelamin A (LA-C661M) and progerin (LA-Δ50). Arrows: BAF nuclear recruitment; arrowheads: HP1α nuclear periphery increase; asterisks: LAP2α nuclear periphery localization. Nuclei were counterstained with DAPI. Bar 10 μm.

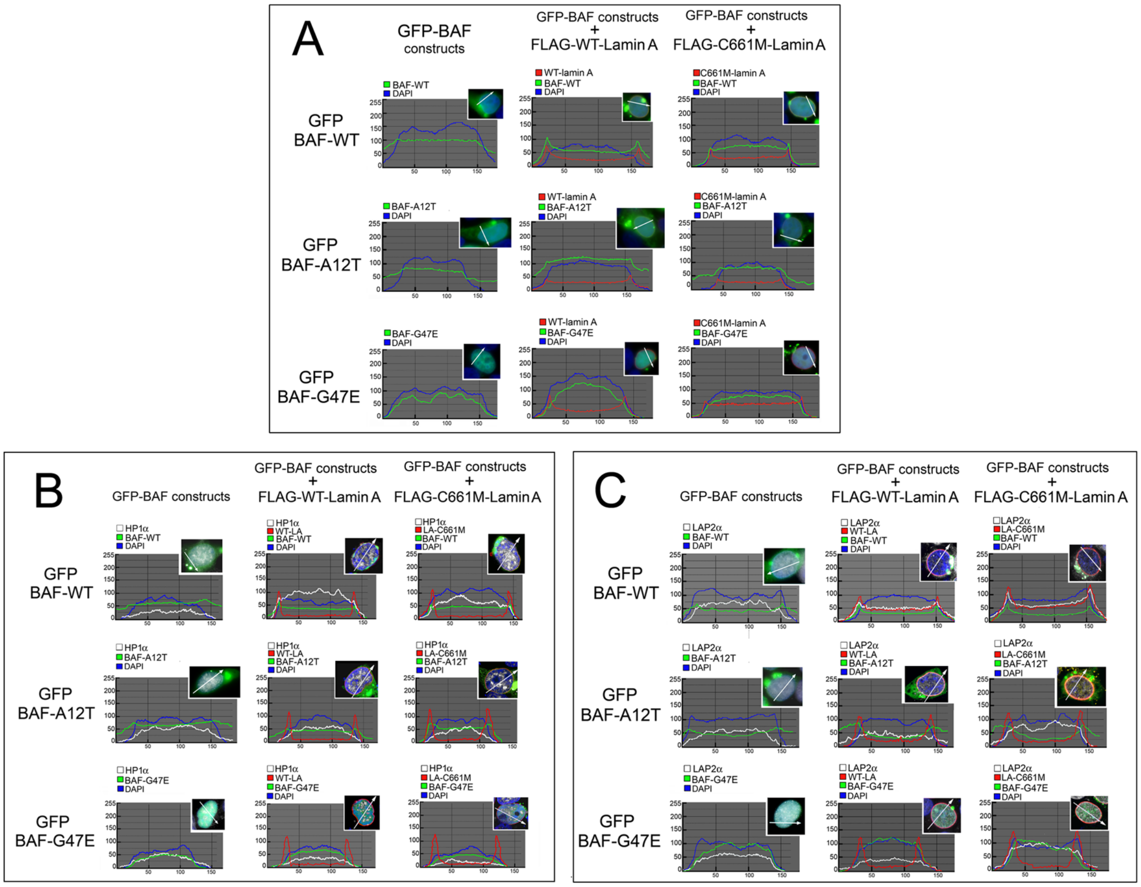

Supplementary Figure S4: Evaluation of GFP-BAF mutants colocalization with FLAG-prelamin A tagged proteins, HP1-alpha and LAP2-alpha in HEK293 cells. (**A**) GFP-BAF constructs colocalization with FLAG-prelamin A constructs. In left graphs line is reported the GFP-BAF constructs staining intensity profiles along the arrow drawn on the cell showed inside each graph. Intensity profiles of GFP-BAF-WT, GFP-BAF-A12T and GFP-BAF-G47E (green) and DAPI (blue) are reported in graphs. In central graph line intensity profiles along arrows drawn on cells showed inside each graph of GFP-BAF mutants (green) , FLAG-lamin A-WT (red) and DNA (DAPI, blue) staining are reported. In the right graphs line intensity profiles of GFP-BAF mutants (green), FLAG-lamin A-C661M (red) and DNA (DAPI, blue) are shown. (**B**) GFP-BAF constructs colocalization with FLAG-prelamin A constructs and HP1*α*. Intensity profiles of GFP-BAF-WT, GFP-BAF-A12T and GFP-BAF-G47E (green), HP1*α* (white) and DNA staining with DAPI (blue) are reported in left line graphs. In central graphs intensity profiles of GFP-constructs (green), FLAG-lamin A-WT (red), HP1*α* and DNA (DAPI, blue) staining measured along arrows droved on the cell showed inside each graph is reported. In the right graphs line the intensity profiles of GFP-BAF constructs (green), FLAG-lamin A-C661M (red), HP1*α* (white) and DNA (DAPI, blue) staining are shown. (**C**) GFP-BAF constructs colocalization with FLAG-prelamin A constructs and LAP2*α*. Intensity profiles of GFP-BAF-WT, GFP-BAF-A12T and GFP-BAF-G47E (green), LAP2*α* (white) and DNA staining with DAPI (blue) are reported in graphs showed in left line. In central graphs intensity profiles of GFP-BAF constructs (green), FLAG-lamin A-WT (red), LAP-alpha (white) and DNA (DAPI, blue) staining measured along arrows droved on the cell showed inside each graph are reported. In the right graphs line intensity profiles of GFP-BAF constructs (green), FLAG-lamin A-C661M (red), LAP2*α* (white) and DNA (DAPI, blue) staining are shown.

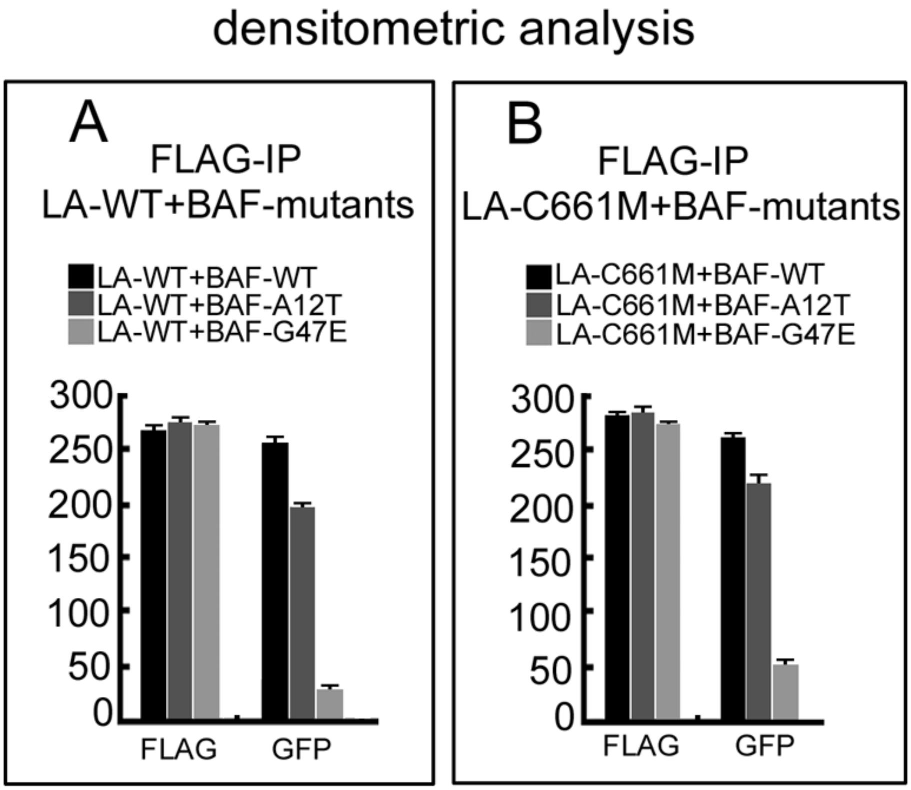

Supplementary Figure S5: Densitometric analysis of coimmunoprecipitation experiments performed in HEK293 transfected cells. Statistical analysis of densitometric bands obtained by Western blotting evaluation of coimmunoprecipitation experiments performed in HEK293 cells expressing FLAG-tagged protein in combination with BAF-WT or BAF-A12T or BAF-G47E. Densitometric analysis of FLAG and GFP immunoblotted bands was performed in triplicate experiments, and the mean values ± S.D. are reported.
